# Repair of Main Pancreatic Duct Injury during Surgery for Congenital Biliary Dilatation in a Child: A Case Report

**DOI:** 10.70352/scrj.cr.26-0244

**Published:** 2026-10-01

**Authors:** Hiroshi Nouso, Takuo Noda, Hiroshi Okano, Terutaka Tanimoto

**Affiliations:** Department of Pediatric Surgery, Okayama University Hospital, Okayama, Okayama, Japan

**Keywords:** congenital biliary dilatation, choledochal cyst, pancreaticobiliary maljunction, pancreatic duct injury, pediatrics

## Abstract

**INTRODUCTION:**

In surgery for congenital biliary dilatation, it is essential to minimize residual intrapancreatic bile duct tissue to prevent future complications, such as intrapancreatic stones and carcinogenesis. However, dissection of the intrapancreatic bile duct is technically demanding and may result in injury to the main pancreatic duct. As a result, very few cases have been reported regarding the management of this complication in pediatric patients. We present a pediatric case of intraoperative main pancreatic duct injury and describe its surgical management.

**CASE PRESENTATION:**

A 3-year-old girl was diagnosed with congenital biliary dilatation. During excision of the intrapancreatic bile duct, a small ductal lumen with clear fluid leakage was identified. Contrast imaging through the ductal lumen revealed the entire main pancreatic duct, confirming the diagnosis of intraoperative pancreatic duct injury. Because the anatomical relationship between the injured site and the distal main pancreatic duct toward the papilla was obscured, repair was undertaken with concern for an accidental division of the pancreaticobiliary junction. A 3-Fr short catheter was inserted into the pancreatic duct as an internal stent to secure pancreatic juice drainage. The injured duct was anastomosed to the remnant intrapancreatic bile duct using 7-0 absorbable sutures, and the repair site was covered with surrounding connective tissue. External drainage was placed adjacent to the repair site. She developed a transient postoperative pancreatic fistula, which resolved with conservative management. No episodes of pancreatitis or pancreatic stone formation were observed during 6 years of follow-up.

**CONCLUSIONS:**

Intraoperative recognition of main pancreatic duct injury and establishment of a secure pancreatic drainage route using an internal stent can lead to favorable outcomes. This case provides practical insight into the management of a rare but serious complication during pediatric surgery for congenital biliary dilatation.

## INTRODUCTION

The standard surgical treatment for congenital biliary dilatation is complete excision of the extrahepatic bile duct followed by hepaticojejunostomy. To prevent future complications, such as intrapancreatic stones and carcinogenesis, minimizing residual intrapancreatic bile duct tissue is essential. With the increasing use of laparoscopic and robot-assisted approaches, surgeons perform meticulous dissection of the bile duct under magnified visualization. However, dissection of the intrapancreatic bile duct remains technically demanding, and its difficulty varies depending on the anatomy of the pancreaticobiliary maljunction and inflammatory changes resulting from previous cholangitis or pancreatitis. Although intraoperative injury to the main pancreatic duct is uncommon, it is a potentially serious complication. Favorable outcomes may be achieved when the injury is recognized promptly and managed appropriately, but inadequate management may lead to postoperative pancreatic fistula, pancreatitis, or other serious postoperative complications.

We report here a pediatric case of intraoperative main pancreatic duct injury that occurred during excision of the intrapancreatic bile duct. The injury was successfully managed by pancreatic duct decompression using an internal stent and peripancreatic drainage, resulting in a favorable postoperative course.

## CASE PRESENTATION

A 3-year-old girl presented with a history of recurrent abdominal pain and nausea and had been treated at a local clinic for suspected cyclic vomiting syndrome. She was admitted to the referring hospital with similar symptoms and was found to have ketotic hypoglycemia. Abdominal ultrasonography was performed because abdominal pain persisted during hospitalization, and it showed dilatation of the common bile duct. She was then referred to our institution for further evaluation and treatment.

Laboratory tests showed elevated pancreatic enzyme levels, with a serum amylase level of 135 U/L and a lipase level of 271 U/L. Contrast-enhanced CT showed cystic dilatation of the common bile duct and mild intrahepatic bile duct dilatation. However, findings suggestive of acute pancreatitis, such as poor pancreatic enhancement or increased peripancreatic fat stranding, were not evident. Magnetic resonance cholangiopancreatography showed a slightly dilated common channel (**Fig. 1A**). However, the pancreaticobiliary junction could not be clearly identified (**Fig. 1B**). On the basis of these findings, congenital biliary dilatation (Todani type Ia) and pancreaticobiliary maljunction were diagnosed. Acute pancreatitis associated with congenital biliary dilatation was suspected as the cause of her abdominal pain. Her symptoms improved promptly with fasting, intravenous fluid therapy, antibiotics, and a protease inhibitor. Definitive surgery for congenital biliary dilatation was scheduled approximately 3 months later.

**Fig. 1:**
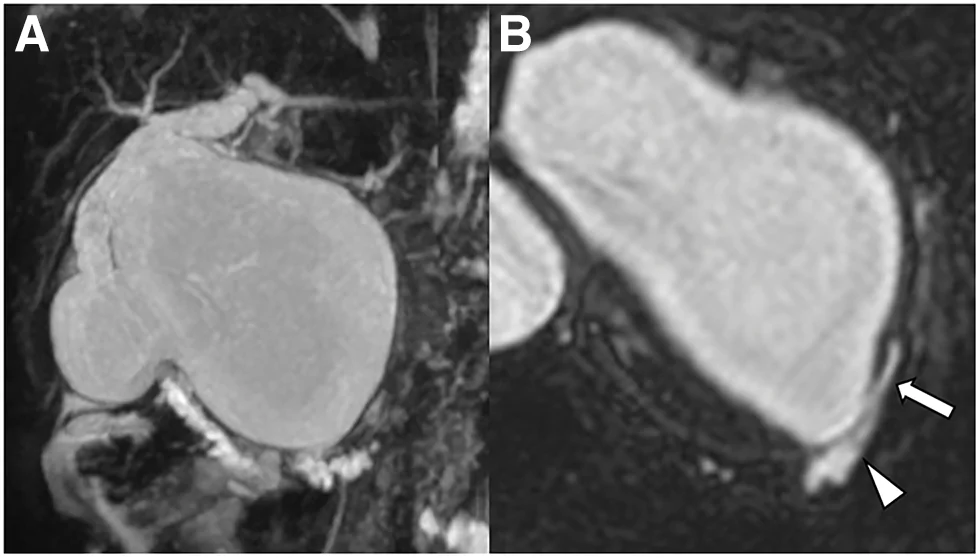
Magnetic resonance cholangiopancreatography. (**A**) MIP image showing the overall configuration from the dilated bile duct to the common channel. (**B**) Enlarged view demonstrating the cystically dilated bile duct and a thin main pancreatic duct (arrow). The presumed junction (arrowhead) is inferred from the close proximity of the bile duct and pancreatic duct. The intrapancreatic bile duct and main pancreatic duct run adjacent to each other over a wide area. MIP, maximum intensity projection

Definitive surgery was performed via a right upper abdominal transverse incision. Intraoperative cholangiography showed that the dilated bile duct narrowed into a common channel and drained into the duodenum. However, the main pancreatic duct was not visualized. After circumferential dissection of the common bile duct within the hepatoduodenal ligament, the anterior wall was incised, and the pancreaticobiliary junction was identified from the luminal side. The intrapancreatic bile duct was dissected down to just above the estimated pancreaticobiliary junction. During this process, a small ductal lumen with clear fluid leakage was identified on the slightly ventral side of the dissection plane at the 3 o’clock position. Contrast injection through the outer cannula of a 24-gauge intravenous catheter inserted into the ductal lumen showed the entire length of the main pancreatic duct in the pancreatic body and tail, leading to a diagnosis of intraoperative main pancreatic duct injury (**Fig. 2A**). Accidental division of the pancreaticobiliary junction was considered because the anatomical relationship between the injured site and the distal main pancreatic duct toward the papilla was unclear (**Fig. 2C**), and repair was then undertaken.

**Fig. 2:**
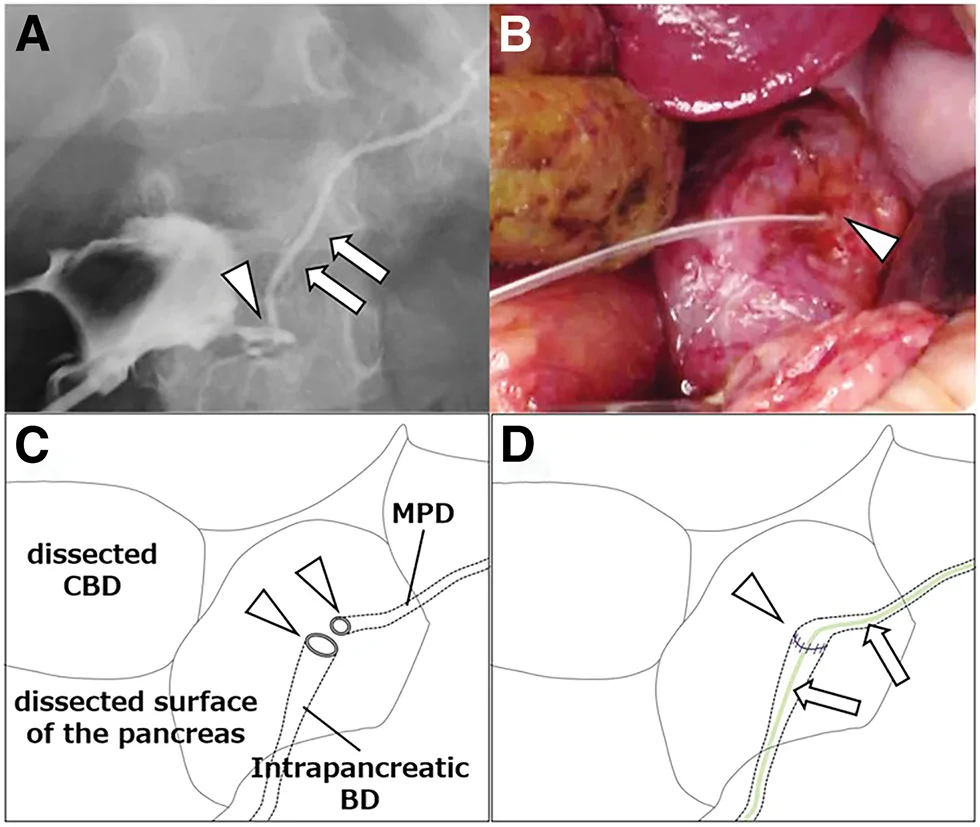
Intraoperative findings. (**A**) Pancreatography via the injured site showing the entire length of the main pancreatic duct (arrows) from the cannulation point of the contrast tube (arrowhead). (**B**) Operative view of the injured main pancreatic duct (arrowhead) identified at the center of a wide pancreatic dissection plane. A 3-Fr multipurpose tube was inserted to temporarily maintain the ductal lumen. (**C**) Schematic illustration before repair. The transected main pancreatic duct and the remnant intrapancreatic bile duct (arrowheads) are exposed at the dissection plane. (**D**) Schematic illustration after repair. An anastomosis was created between the injured main pancreatic duct and the remnant intrapancreatic bile duct (arrowhead). A 3-Fr internal stent (arrows) was placed across the anastomosis into the duodenum to maintain pancreatic drainage. BD, bile duct; CBD, common bile duct; MPD, main pancreatic duct

A short segment of a 3-Fr multipurpose tube (Atom Medical, Tokyo, Japan) was placed as an internal stent to facilitate spontaneous dislodgement (**Fig. 2B**). The tube was inserted approximately 1.5 cm into the proximal main pancreatic duct through the injured site, and its passage into the duodenum via the intrapancreatic bile duct was confirmed under fluoroscopy. An anastomosis between the injured main pancreatic duct and the remnant intrapancreatic bile duct was created using interrupted 7-0 polydioxanone sutures to secure pancreatic juice drainage (**Fig. 2D**). To localize potential pancreatic fistula in the event of anastomotic failure, surrounding connective tissue at the pancreatic dissection plane was approximated to cover the repair site. After repair of the pancreatic duct, complete excision of the extrahepatic bile duct and biliary reconstruction with hepaticojejunostomy were performed as planned. A 15-Fr Blake drain (Ethicon, Somerville, NJ, USA) was placed adjacent to the pancreatic duct repair site and loosely fixed to the surrounding connective tissue with absorbable sutures to prevent displacement during subsequent procedures. An additional drain of the same type was placed posterior to the hepaticojejunostomy, and the abdomen was closed. The total operative time was 8 hours and 34 minutes.

On POD 1, the serum amylase level was elevated to 629 U/L, and the amylase level in the drainage fluid adjacent to the repair site reached 13348 U/L, consistent with a Grade A postoperative pancreatic fistula. This elevation was managed conservatively, and enzyme levels gradually decreased to 91 U/L in the serum and 327 U/L in the drainage fluid by POD 6. Oral intake was resumed on POD 7, and no abdominal symptoms, inflammatory response, or further elevation of pancreatic enzymes was observed. The drains were removed on POD 16, and the patient was discharged on POD 17. A follow-up radiograph obtained on POD 38 confirmed spontaneous dislodgement of the stent, and no stent-related complications were observed. At the most recent follow-up, 6 years after surgery, the patient remained asymptomatic, with no evidence of pancreatitis or pancreatic stone formation.

## DISCUSSION

Complete excision of the extrahepatic bile duct remains the standard surgical treatment for congenital biliary dilatation and is recommended to prevent future complications under the current Japanese guidelines.^1,2)^ Dissection of the intrapancreatic bile duct is one of the most critical steps in this procedure because any residual bile duct tissue may lead to recurrent pancreatitis, stone formation, and carcinogenesis.^3,4)^ Ando et al. emphasized the importance of identifying the appropriate dissection plane along the epicholedochal plexus.^5)^ However, this plane may become obscure because of inflammatory changes related to previous cholangitis or pancreatitis, or because of compression from a cystically dilated bile duct. Under such conditions, dissection becomes technically challenging, thereby increasing the risk of inadvertent injury to the main pancreatic duct.

Although intraoperative injury to the main pancreatic duct is uncommon, it may lead to serious postoperative complications, including pancreatic fistula and pancreatitis. Reports describing this complication remain limited, particularly in pediatric patients.^6,7)^ Previous reports have described a variety of management strategies, including primary repair with pancreatic duct stenting, external drainage, and delayed reconstructive procedures.^6–10)^ (**Table 1**) Furthermore, no systematic strategy for managing this complication has been established, and intraoperative decision-making is largely dependent on the surgeon’s judgment.

**Table 1:** Previously reported cases of intraoperative pancreatic duct injury

Author	Age	Procedure	Timing	Mechanism of injury	Repair	Complications
Mori et al.^6)^	6 mo	Open	Intraoperative	Obscured dissection planes	Primary repair + external stent	
Kato et al.^7)^ (Case 1)	2 y	Laparoscopic	Intraoperative	Obscured dissection planes	Primary repair + ERPD	
Kato et al.^7)^ (Case 2)	8 y	Laparoscopic	Intraoperative	Obscured dissection planes anomalous course of MPD	Primary repair + ERPD	POPF
Ray et al.^8)^	23 y	Open	Delayed	Inadvertent ligation of MPD	Pancreaticogastrostomy	MPD dilatation
Choi et al.^9)^	55 y	Open	Intraoperative	Intrapancreatic BD avulsion caused by strong traction	Endoscopic duodenocystostomy	POPF type 2 diabetes
Present case	3 y	Open	Intraoperative	Obscured dissection planes	Primary repair + internal stent	POPF

BD, bile duct; ERPD, endoscopic retrograde pancreatic drainage; MPD, main pancreatic duct; POPF, postoperative pancreatic fistula

In our case, several factors likely contributed to the occurrence of main pancreatic duct injury. Intraoperative cholangiography did not clearly demonstrate the main pancreatic duct, which may have led to an underestimation of the level of the pancreaticobiliary junction. Additionally, inflammatory changes due to preceding pancreatitis likely obscured the optimal dissection plane. Moreover, the main pancreatic duct may have been displaced and closely adherent to the bile duct because of compression. Therefore, identifying the course of the pancreatic duct as accurately as possible using preoperative imaging and maintaining constant awareness of its relationship to the dissection plane during surgery are important. If the anatomical relationship between the bile duct and pancreatic duct remains unclear despite intraoperative cholangiography and choledochoscopy, surgeons should consider the possibility that the pancreaticobiliary junction is located more proximally than anticipated. Additionally, when inflammation obscures the epicholedochal plexus, particular caution is warranted during distal dissection. In retrospect, a more conservative distal dissection approach with attention to a potentially displaced pancreatic duct might have reduced the risk of injury in our case.

The Japanese clinical practice guidelines for pancreaticobiliary maljunction and congenital biliary dilatation describe repair of the injured duct using 6-0 absorbable sutures combined with a thin transpapillary pancreatic duct stent for decompression.^11)^ Similar management has been reported with favorable outcomes in pediatric patients.^6,7)^ The primary objectives of treatment are to preserve the ductal lumen and establish an adequate drainage route for pancreatic juice.

In our case, the injured duct and the remnant intrapancreatic bile duct remained closely approximated, allowing reconstruction without tension. Therefore, we selected reconstruction by an anastomosis between the injured main pancreatic duct and the remnant intrapancreatic bile duct. A relatively wide anastomotic lumen could be created between the injured main pancreatic duct and the remnant intrapancreatic bile duct, which was expected to provide reliable pancreatic drainage and effective ductal decompression. A short segment of the thinnest and most flexible catheter available was used as an internal stent to maintain ductal patency. The surrounding connective tissue was used to reinforce the repair, and an external drain was placed adjacent to the anastomosis to control potential leakage.

Alternative reconstructive options may be considered depending on the extent of injury, including external pancreatic duct drainage and pancreaticojejunostomy. External drainage can provide effective decompression and allow direct monitoring of pancreatic juice output, but prolonged drainage through a long, narrow-diameter catheter may increase the risk of tube obstruction. Pancreaticojejunostomy may be considered in cases of extensive ductal disruption. However, this procedure should preferably be avoided in infants because a small duct diameter makes it technically demanding. The favorable long-term outcome, including the absence of recurrent pancreatitis and pancreatic stone formation during 6 years of follow-up in our case, supports the validity of this reconstructive strategy.

The management of intraoperative main pancreatic duct injury requires case-specific judgment based on the operative findings and the anatomical relationship at the injury site. Although main pancreatic duct injury during pediatric surgery for congenital biliary dilatation is rare, the accumulation of detailed clinical experience is essential. The present case suggests that reconstruction between the injured main pancreatic duct and the remnant intrapancreatic bile duct can be a feasible option when tension-free repair is achievable. Four points appear to be particularly important in this setting: prompt intraoperative recognition, selection of an appropriate reconstruction strategy that preserves pancreatic drainage, secure decompression of pancreatic juice flow with internal stenting, and strategic placement of external drainage to control potential leakage.

## CONCLUSIONS

Intraoperative recognition of main pancreatic duct injury and establishment of a secure pancreatic drainage route using an internal stent can lead to favorable outcomes. This case provides practical insight into the management of an uncommon but potentially serious complication during pediatric surgery for congenital biliary dilatation. Awareness of this potential complication is essential for pediatric surgeons performing intrapancreatic bile duct dissection.

## DECLARATIONS

### Availability of data and materials

All data generated or analyzed during this study are included in this published article.
